# Supplementary material for: Contrast-enhanced computed tomography findings of canine primary renal tumors including renal cell carcinoma, lymphoma, and hemangiosarcoma
Source: PLoS One. 2019 Nov 22;14(11):e0225211. doi: 10.1371/journal.pone.0225211 (PMC6874336; doi:10.1371/journal.pone.0225211)
Supplement: Attachment — Submitted filename: response to reviewersPLOS ONE kidney 2nd 20190906.docx [file pone.0225211.s002.docx]

Dear Douglas H. Thamm, V.M.D.

We edited this manuscript to correct grammatical, spelling or wording errors by a native English speaker.

I am returning herewith a manuscript revised according to reviewer's comments. I revised our manuscript with letters with highlights.

I hope that revised manuscript is now acceptable for publication.

Yours sincerely,

Toshiyuki Tanaka,

Department of Veterinary Surgery,

School of Life and Environmental Sciences,

Osaka Prefecture University,

1-58 Rinku-ouraikita, Izumisano,

Osaka 598-8531,

Japan

**Dear reviewer1**

According to your review, we think this manuscript submit.

Our main changes are:

There are still some significant grammatical errors that need to be addressed. For example:

L20: We changed “for the RANK” to “to rank”.

We changed “remarkable” to “severe” in this manuscript.

At L35, L164, L169, L388, L390

L308: “is the absence of lack of necrosis,” is double negative. Sorry, we changed to “is the absence of necrosis,”.

Etc…..

→ We edited this manuscript to correct grammatical, spelling or wording errors by a native English speaker.

There are still some specific concerns I have, which are listed as follows:

Line 23: instead of “consequently” consider wording such as “In this retrospective study” so that it is clear in the abstract that is what type of study this is.

→We changed “consequently” to “In this retrospective study”.

Line 29: Lymphoma should not be plural here.

→We changed “lymphomas” to “lymphoma”.

L44: This is the wrong reference. That particular “what’s your diagnosis” is regarding HOD in a dog. I would also like to state that your reference formatting for this and other articles lists the volume but not the issue which can make it difficult to locate an article. In particular for this paper, which did not come up in a Google scholar search, pages aren’t listed on the JAVMA website which made it hard to find. Also if you are going to report the incidence of a tumor that reference should not be from a “What’s your diagnosis” article. I assume there is an actual reference that is quoted in this paper which is the one that should be used.

→ We changed reference. “Deborah WK and Sarah KM. Tumors of the urinary system In: Vail DM, editor. *Withrow and MacEwen’s small animal clinical oncology*. 5th ed. St Louis: Saunders Elsevier, 2012; p.p. 579.”.

Line 81: take out “originating from another tumor or multiple tumors" as that is the definition of metastatic
→We deleted “originating from another tumor or multiple tumors”.

Line 126: add ‘reported’ for the normal range

→

We added “reported”. So, we changed “the normal range” to “the reported normal range”.

Line 127: change to “lung mets was determined from the thoracic portion of the the CT examined in a detailed, lung window”

→

We changed “lung metastasis was determined from the lung lesion enhancement in the post-contrast phase.” to “lung metastasis was determined from the thoracic portion of the CT examined in a detailed, lung window”.

Line 153: I’d report the number of tumors in each category at the start of this paragraph then the breakdown
→

We changed “All renal tumor diagnoses were confirmed by cytology or histopathology. Finally, tumors were diagnosed as RCC (n = 9, 60%), lymphoma (n = 4, 27%), and HSA (n = 2, 13%).” to “All renal tumor diagnoses were diagnosed as RCC (n = 9, 60%), lymphoma (n = 4, 27%), and HSA (n = 2, 13%) by cytology or histopathology.”.

Line 185: saying RCC’s had no BV enhancement on certain phases in the body with the table above it saying 89% had vessel enhacement is confusing
→

We changed the sentence

“In RCC cases, vessel enhancement was not detected in the nephrographic or excretory phases (Fig 2).” to “In RCC cases, vessel enhancement was detected only in the corticomedullary phase (Fig 2).”

Lines 212-234: in your discussion you never discuss what the significance of having a meaningful effect size or the results of the post hoc test being significant means. If you are not going to explain these results to the reader, please exclude. In line 238 you say meaningful effect size was detected but need more of an explanation for this
→

L241-244: We explained about effect size. And added new references. We added the sentence ”P-value is dependent on the sample size. Therefore, small sample size have a potential type II error. An effect size is independent of sample size, and indicate the magnitude or derection between variables. Further study about attenuation values between each tumors is needed in large sample size.”

Line 249: while it is obvious, please include brief description for excretory phase here also.

→ We added the sentence “Excretory phase is excretion of urine after 60s after contrast injection.”.

Line 261, 349, 355: be careful to state that findings in this study were not significant

→

L261: Here, we described that statistical analysis was not performed. So, we changed the sentence “Our CT study also showed similar findings in RCC cases” to “Although statistical analysis was not performed, our CT study also showed similar findings in RCC cases.”.

L349: we added that there was no significant difference of attenuation values between HSA and other renal tumors.

So, we added the sentence “Although there was no significant difference,”

L355: We thought that this sentence described about further study. Therefore, we keep the sentence in an unchanged form.

Line 277-278: I assume you mean the masses created by the RCC and LSA?

→ This study include HSA. So, we added HSA.

We changed the sentence to ”In this study, RCC, lymphoma and HSA showed lower attenuation compared to the renal cortex in each phase.”.

Line 285: “renal fna can be used to assist with the cytological diagnosis of”
→

We changed the sentence “renal FNA can also diagnose solitary …” to “renal FNA can be used to assist with the cytological diagnosis of solitary …”.

Line 327-330: remove, you didn’t include metastatic lesions so no need to include in discussion

→

L327: We removed the sentence” Metastatic disease, which commonly originates from breast or lung primary tumors, causes bilateral, multifocal renal masses [32]. Unfortunately, our study excluded cases with metastatic lesions. Therefore, further research is needed to examine the differences between canine renal lymphoma and metastatic lesions on CT.”

Line 346-348: I’d remove the description of human angiosarcomas after saying they have irregular vascular spaces or channels.
→

We changed the sentence “In humans, primary renal angiosarcomas have multiple, irregular connected vascular spaces or channels, which are formed by discrete and large endothelial cells with several degrees of cytological pleomorphism, nuclear atypia, mitotic activity, and multilayering [42]. Additionally, human renal angiosarcomas have a variety of epithelioid and spindle cell morphologies [42].” to “In humans, primary renal angiosarcomas have multiple, irregular connected vascular spaces or channels . Additionally, human renal angiosarcomas have a variety of epithelioid and spindle cell morphologies.”

Line 367: why do you think this is true? You only had 1 dog that had RCC and lymphadenopathy

→ We changed the sentence “The lymph metastasis sites of dogs with RCC are similar to those of humans with RCC [44].” to “ The lymph metastasis sites of dogs with RCC may be similar to those of humans with RCC. However, Lymphadenopathy of RCC was only one case in this study. Thus, further research is needed.”.

Line 373: add “may be”

→

Changed “infiltration is important.” to “infiltration may be important.”.

Line 381: add “possible”

→

We added “possible”. So, “lung metastasis” to “possible lung metastasis”.

# I hope that the revised paper meets your approval and will be more suitable for publication in PLOS ONE.

Yours sincerely,

Toshiyuki Tanaka
