# Supplementary material for: Contrast-enhanced computed tomography findings of canine primary renal tumors including renal cell carcinoma, lymphoma, and hemangiosarcoma
Source: PLoS One. 2019 Nov 22;14(11):e0225211. doi: 10.1371/journal.pone.0225211 (PMC6874336; doi:10.1371/journal.pone.0225211)
Supplement: Attachment — Submitted filename: response to reviewers PLOS ONE kidney 3rd 20191017.docx [file pone.0225211.s003.docx]

Dear Douglas H. Thamm, V.M.D.

I am returning herewith a manuscript revised according to reviewer's comments. I revised our manuscript with letters with highlights.

I hope that revised manuscript is now acceptable for publication.

Yours sincerely,

Toshiyuki Tanaka,

Department of Veterinary Surgery,

School of Life and Environmental Sciences,

Osaka Prefecture University,

1-58 Rinku-ouraikita, Izumisano,

Osaka 598-8531,

Japan

**Dear reviewer1**

According to your review, we think this manuscript submit.

Our main changes are:

#37that states most lymphoma cases were unilateral I think is misleading. There are many references saying the majority of renal LSA cases in dogs are bilateral, much similar to your study. Look for Taylor, A. et al, JSAP 2019; Taylor, A, et al. VRU 2014
→

L329: We described about RCC in lymphoma paragraph. SO, we deleted “In canine RCC, 96 % of tumors were unilateral, and 4 % were bilateral [37].”.

I think there was a misunderstanding regarding my comments about effect size. If you are going to say in results it was meaningful, as was post hoc testing being meaningful between tumor types, I think a discussion point would be what this means – that maybe there would be significant differences if you had larger sample size? Or that you truly think this is meaningful/significant? Please make clear what this meant to you.

→

We think large effect size show not significant difference, but tend to be different. We used effect size as objective index of difference.

Line 154-155 should be at the start of the paragraph

→

L148: We moved the sentence “All renal tumors were diagnosed as RCC (n = 9, 60 %), lymphoma (n = 4, 27 %), or HSA (n = 2, 13 %) through cytology or histopathology.” to the start of the paragraph.

Table 1 – should say “Presumed” lung metastasis

→

We changed “lung metastasis” to “presumed lung metastasis” at table 1.

Line 277-278 – I assume the lower HU’s in comparison to the renal cortex means that the masses in the individual dogs compared to the renal cortex in same imaging study and NOT compared to the quoted values in the start of this paragraph?
→

L277-278: We think so. In this paragraph, we did not describe about HSA. So, we deleted HSA. So, we changed the sentence “RCC, lymphoma, and HSA showed lower attenuation compared to the renal cortex in each phase.” to “In this study, RCC and lymphoma showed attenuations lower than 349.4 ± 65.3 HU in the corticomedullary phase. ”

Still the occasional wording/grammar errors.

→

Again, we edit this manuscript to correct grammatical, spelling or wording errors by a native English speaker.

# I hope that the revised paper meets your approval and will be more suitable for publication in PLOS ONE.

Yours sincerely,

Toshiyuki Tanaka
